# Supplementary material for: Clinical evaluation of the alcohol use disorders identification test (AUDIT) in Moshi, Tanzania
Source: PLoS One. 2023 Nov 8;18(11):e0287835. doi: 10.1371/journal.pone.0287835 (PMC10631671; doi:10.1371/journal.pone.0287835)
Supplement: S1 File — Study Information & Consent Form (English): Study Information & Consent Form (Swahili): AUD Screening questions–DSM-V (English): AUDIT (English):AUD Screening questions–DSM-V (Swahili): AUDIT (Swahili). (DOCX) [file pone.0287835.s001.docx]

**Supporting Information**

**SUPPLEMENTARY TABLE**

**Table S1:** AUDIT screening parameters.

| **AUDIT Score** | **n** | **Sensitivity** | **Specificity** | **PPV** | **NPV** |
| --- | --- | --- | --- | --- | --- |
| **1** | 40 | 1.00 | 0.00 | 0.36 | NaN |
| **2** | 17 | 0.99 | 0.23 | 0.42 | 0.98 |
| **3** | 19 | 0.97 | 0.32 | 0.44 | 0.95 |
| **4** | 18 | 0.97 | 0.44 | 0.49 | 0.96 |
| **5** | 18 | 0.91 | 0.51 | 0.51 | 0.91 |
| **6** | 21 | 0.86 | 0.59 | 0.54 | 0.88 |
| **7** | 18 | 0.83 | 0.70 | 0.60 | 0.88 |
| **8** | 11 | 0.77 | 0.78 | 0.66 | 0.86 |
| **9** | 11 | 0.73 | 0.82 | 0.69 | 0.85 |
| **10** | 8 | 0.68 | 0.86 | 0.73 | 0.83 |
| **11** | 12 | 0.65 | 0.89 | 0.77 | 0.82 |
| **12** | 8 | 0.59 | 0.93 | 0.82 | 0.80 |
| **13** | 7 | 0.52 | 0.94 | 0.83 | 0.78 |
| **14** | 8 | 0.49 | 0.96 | 0.88 | 0.77 |
| **15** | 9 | 0.43 | 0.98 | 0.93 | 0.76 |
| **16** | 2 | 0.35 | 0.99 | 0.94 | 0.73 |
| **17** | 5 | 0.33 | 0.99 | 0.94 | 0.73 |
| **18** | 1 | 0.28 | 0.99 | 0.96 | 0.72 |
| **19** | 2 | 0.27 | 0.99 | 0.96 | 0.71 |
| **20** | 3 | 0.25 | 0.99 | 0.96 | 0.71 |
| **21** | 2 | 0.22 | 0.99 | 0.95 | 0.70 |
| **22** | 3 | 0.20 | 0.99 | 0.95 | 0.69 |
| **23** | 2 | 0.16 | 0.99 | 0.94 | 0.68 |
| **24** | 2 | 0.14 | 0.99 | 0.93 | 0.68 |
| **25** | 3 | 0.12 | 0.99 | 0.92 | 0.67 |
| **26** | 1 | 0.09 | 0.99 | 0.89 | 0.66 |
| **27** | 0 | - | - | - | - |
| **28** | 1 | 0.08 | 0.99 | 0.88 | 0.66 |
| **29** | 1 | 0.08 | 1.00 | 1.00 | 0.66 |
| **30** | 1 | 0.07 | 1.00 | 1.00 | 0.66 |
| **31** | 1 | 0.05 | 1.00 | 1.00 | 0.66 |
| **32** | 2 | 0.04 | 1.00 | 1.00 | 0.65 |
| **33** | 1 | 0.02 | 1.00 | 1.00 | 0.65 |
| **34** | 1 | 0.01 | 1.00 | 1.00 | 0.65 |

*Note: Bolded numbers represent the maximal Youden index*

*Abbreviations: NPV, negative predictive value; PPV, positive predictive value; Sens, Sensitivity; Spec, Specificity.*

**Study Instruments**

**Study Information & Consent Form (English):**

**INTRODUCTION**

You are being asked to take part in this research study as a member of the general public. This study is being conducted by Dr. Mark Mvungi, Acting Director of Hospital Services, Dr. Blandina Mmbaga Kilimanjaro Christian Medical Center and Dr. Catherine Staton of the Division of Emergency Medicine at Duke University.

Research studies are voluntary and include only people who choose to take part.  Please read this consent form carefully and take your time making your decision. As your study staff member discusses this consent form with you, please ask him/her to explain any words or information that you do not clearly understand. The nature of the study, risks, inconveniences, discomforts, and other important information about the study are listed below. You are free to ask questions about this study at any time. If you agree to take part in this study, you will be asked to sign and date this consent form. You will get a copy to keep.

**WHY IS THIS STUDY BEING DONE?** The purpose of this study to determine if survey questions that are used elsewhere in the world about alcohol work well here in Tanzania as well.

**WHAT DO I HAVE TO DO IF I AM IN THIS STUDY?**

If you agree to participate, we will ask you to answer some questions about your lifestyle and behavior, and any injuries you might have suffered. Our questionnaire will take about 30 minutes of your time.

**HOW MANY PEOPLE WILL TAKE PART IN THIS STUDY?**

About 350 people will participate in this study.

**HOW LONG WILL I BE IN THIS STUDY?**

Most likely your survey will last about 30 minutes.

**WHY WOULD THE DOCTOR TAKE ME OFF THIS STUDY EARLY?**

The study could be ended early by the Ministry of Health in Tanzania or by the Ethics Committee of KCMC. Ethics Committees and Institutional Review Boards watch over the safety and rights of research subjects. Also, the study could be ended early by the following groups in the United States, the Duke University Health System Institutional Review Board, the National Institutes of Health, and the Office of Human Research Protections.

**WHAT ARE THE RISKS AND BENEFITS OF THE STUDY?**

There is no benefit to you for participating in this study. There are no physical risks associated with this study. There is, however, the potential risk of loss of confidentiality. Every effort will be made to keep your information confidential; however, this cannot be guaranteed. Some of the questions we will ask you as part of this study may make you feel uncomfortable. You may refuse to answer any of the questions and you may take a break at any time during the study. You may stop your participation in this study at any time.

If your answers to questions show that you need further medical or social assistance, we are obligated to, and will, refer you to an appropriate medical professional for treatment. We will not pay for the costs of your medical treatment or transport costs associated with these additional treatments.

**CONFIDENTIALITY**

Study records will be kept confidential as required by law. Your records will be assigned a unique study number. Information that links your name to the study number will be kept in a locked cabinet that can only be accessed by members of the research team. No personal identifiers will be sent to or used at Duke. If information from this study is presented at scientific meetings or in scientific journals, your identity will not be revealed.

**WHAT ARE THE COSTS TO ME?**

There is no additional cost to you for taking part in this research study.

**WILL I RECEIVE ANY PAYMENTS?**

There will be no financial compensation for participating in this study.

**WHAT ABOUT RESEARCH RELATED INJURIES?**

Immediate necessary care and support is available if an individual is injured because of participation in this research project, however, there is no provision for free medical care or for monetary compensation for such an injury. For questions about the study or research-related injury, contact Dr. Mark Mvungi from KCMC at (255) 78 62 40 988 or at (255) 75 43 01 149

**VOLUNTARY PARTICIPATION/RIGHT TO WITHDRAW**

You may choose not to be in the study, or, if you agree to be in the study, you may withdraw from the study at any time. If you agree to participate, you may refuse to answer any question or stop the interview at any time. Your decision not to participate or to withdraw from the study will not involve any penalty or loss of benefits, and will not affect your access to health care.

Your decision to not participate or to withdraw from the study will not involve any penalty or loss of benefits to which you are entitled, and will not affect your access to health care at KCMC.  If you do decide to withdraw, we ask that you contact Dr. Mark Mvungi in writing and let him know that you are withdrawing from the study. His mailing address is KCMC-Duke Collaboration, Box 3010, Sokoine Road, Moshi.

We will tell you about new information from this or other studies that may affect your health, welfare or willingness to stay in this study. If you want the results of the study, let the study staffs know.

**WHAT DO I DO IF I HAVE QUESTIONS OR PROBLEMS?**

For questions about the study or a research-related injury, or if you have complaints, concerns or suggestions about the research, contact Dr. Mark Mvungi from KCMC at (255) 78 62 40 988 or at (255) 75 43 01 149/ For questions about the study or research-related injury, contact Dr. Mark Mvungi from KCMC at (255) 78 62 40 988 or at (255) 75 43 01 149

For questions about your rights as a research participant, or to discuss problems, concerns or suggestions related to the research, or to obtain information or offer input about the research, contact the Kilimanjaro Christian Medical Centre (KCMC) Ethics Committee at telephone number (255) 27 27-53909 or the Duke University Health Systems Institutional Review Board at +1-919-668-5111.

**STATEMENT OF CONSENT**

"The purpose of this study, procedures to be followed, risks and benefits have been explained to me.  I have been allowed to ask the questions I have, and my questions have been answered to my satisfaction.  I have been told whom to contact if I have additional questions.  I have read this consent form and agree to be in this study with the "understanding that I may withdraw at any time.  I have been told that I will be given a signed and dated copy of this consent form to keep."

Participant’s Name (Print)   Participant’s Signature and Date

**Study Information & Consent Form (Swahili):**

**UTANGULIZI**

Unaombwa kushiriki katika utafiti huu kwa sababu umetibiwa au kuna mwanafamilia aliwahi kutibiwa katika kitengo cha dharura cha hospitali ya KCMC kwa matatizo ya kuumia. Utafiti huu unafanywa na Daktari Mark Mvungi, Kaimu Mkurugenzi wa huduma za hospitali ya KCMC, Daktari Blandina Mmbaga wa KCMC na Daktari Catherine Staton wa Idara ya matibabu ya dharura katika chuo kikuu cha Duke.

Research studies are voluntary and include only people who choose to take part.  Please read this consent form carefully and take your time making your decision. As your study staff member discusses this consent form with you, please ask him/her to explain any words or information that you do not clearly understand. The nature of the study, risks, inconveniences, discomforts, and other important information about the study are listed below. You are free to ask questions about this study at any time. If you agree to take part in this study, you will be asked to sign and date this consent form. You will get a copy to keep.

**KWA NINI UTAFITI HUU UNAFANYIKA?**

Lengo la utafiti huu ni kuonesha kama maswali ya utafiti yanayotumika sehemu nyingine duniani kuhusu pombe yanafanya vizuri na hapa Tanzania pia.

**NINATAKIWA KUFANYA NINI KAMA NINAHUSIKA KWENYE UTAFITI HUU?**

Kama unakubali kushiriki, tutakuomba kujibu baadhi ya maswali kuhusu mfumo wa maisha yako na tabia, na majeraha yoyote utakayokuwa umeumia. Dodoso letu litachukuwa kama dakika 30 za muda wako.

**NI WATU WANGAPI WATASHIRIKI KATIKA UTAFITI HUU?**

Ni kama watu 350 watashiriki katika utafiti huu .

**NTASHIRIKI KATIKA UTAFITI KWA MUDA GANI?**

Sana sana utafiti utachukuwa kama dakika 30 hivi.

**KWA NINI DAKTARI ANAWEZA KUNIONDOA KWENYE UTAFITI HUU MAPEMA?**

Utafiti unaweza kumalizwa mapema na Wizara ya Afya ya Tanzania au Kamati ya Maadili ya KCMC. Kamati ya maadili na Taasisi ya bodi ya mapitio vinaangalia usalama na haki za mhusika wa utafiti. Pia, utafiti unaweza kusitishwa mapema na makundi yafuatayo ya Amerika, Bodi ya upitiaji wa mfumo wa afya ya Taasisi ya chuo kikuu cha Duke,Taasisi ya taifa ya afya, na ofisi ya kulinda tafiti za  kibinadamu.

**ZIPI NI HATARI NA FAIDA ZA UTAFITI?**

Hakuna faida yoyote kwa wewe kushiriki katika utafiti huu. Hakuna hatari yoyote ya kimwili inayohusika na utafiti huu. Hata hivyo, kuna hatari ya upotevu wa usiri. Kila jitihada zitafanyika kuhifadhi taarifa zako kwa usiri; hata hivyo, hii haiwezi kudhaminiwa. Baadhi ya maswali tutakayokuuliza kama sehemu ya utafiti huu yatakufanya uhisi wasiwasi. Unaweza kukataa kujibu maswali yoyote na unaweza kuchukua mapumziko muda wowote wakati wa utafiti huu. Unaweza kusitisha ushiriki wako kwenye utafiti huu wakati wowote.

Kama majibu yako yanaonesha kwamba unahitaji msaada zaidi wa matibabu au wa kijamii, sisi tuna wajibu wa kukurejesha kwa mtaalamu sahihi wa matibabu. Hatutalipa gharama za matibabu yako au gharama za usafiri zinazo husiana na matibabu haya ya ziada.

**USIRI**

Kumbukumbu za utafiti zitahifadhiwa kwa usiri kama sheria zinavyotaka. Kumbukumbu zako zitapewa nambari ya pekee ya utafiti. Taarifa zinazohusisha jina lako na nambari ya utafiti vitawekwa kwenye kabati linalofungwa ambalo litatumiwa na wahusika wa timu ya utafiti tu. Hakuna vitambulisho binafsi ambavyo vitatumwa au kutumika na Duke. Kama taarifa kutoka katika utafiti huu zitawakilishwa kwenye mikutano yoyote ya kisayansi au kwenye majarida ya kisayansi,utambulisho wako hautoonyeshwa.

**ZIPI GHARAMA KWANGU?**

Hakuna gharama zozote za ziada kwa wewe kushiriki kwenye utafiti huu.

**NITAPATA MALIPO YOYOTE?**

Hakutakuwa na fidia yoyote ya kifedha kwa kushiriki katika utafiti huu.

**VIPI KUHUSU MAJERAHA YANAYOHUSISHA UTAFITI?**

Huduma muhimu na msaada wa haraka upo endapo mtu ameumia kwa sababu ya kushiriki kwenye mradi huu wa utafiti, hata hivyo, hakuna uotaji wa huduma ya matibabu ya bure au kufidia pesa kwa majeraha hayo.

Kwa maswali kuhusu majeraha yanayohusika na utafiti au uchunguzi, wasiliana na Daktari Mark Mvungi kutoka KCMC kwa namba 0786240988 au kwa 0754301149.

**HIYARI YA USHIRIKI/HAKI YA KUJITOA**

Unaweza kuchagua kutokuwepo kwenye utafiti, au, kama unakubali kuwa kwenye utafiti, unaweza kujitoa kwenye utafiti muda wowote. Kama unakubali kushiriki, unaweza kukataa kujibu swali lolote au kusimamisha mahojiano katika muda wowote. maamuzi yako ya kutoshiriki au kujitoa kwenye utafiti hayatahusisha adhabu yoyote au kupoteza faida, na haitokuathiri wewe kupata huduma ya matibabu.

Maamuzi yako ya kutokushiriki au kujitoa kutoka kwenye utafiti hayatahusisha adhabu yoyote au kupoteza faida ambayo ni haki yako, na haita athiri upataji wako wa huduma ya matibabu hapa KCMC.  Kama unaamua kujitoa, Tunakuomba uwasiliane na Daktari Mark Mvungi kwa maandishi na kumjulisha kwamba unajitoa kwenye utafiti. Anuani yake ya barua ni KCMC-Duke collaboration, Box 3010, Sokoine Road, Moshi.

Tutakutaarifu kuhusu taarifa mpya kutoka kwenye utafiti huu au tafiti nyingine ambazo zinaweza kuathiri afya yako, ustawi au matakwa ya kuwepo kwenye utafiti huu. Kama unahitaji matokeo ya utafiti huu, wajulishe watumishi wa utafiti.

**NIFANYE NINI KAMA NINA MASWALI AU MATATIZO?**

Kwa maswali kuhusu Utafiti au majeraha yanayohusisha utafiti, au kama una malalamiko, wasiwasi, au mapendekezo kuhusu utafiti, wasiliana na Daktari Mark Mvungi kutoka KCMC kwa namba 0786240988 au kwa 0754301149.

Kwa maswali kuhusu haki zako kama mshiriki wa utafiti, au kujadili matatizo, wasiwasi, au mapendekezo yanayohusu utafiti, au kupata taarifa au kutoa mchango kuhusu utafiti, wasiliana na kamati ya maadili ya KCMC kwa namba ya simu (255) 27 27-53909 au Bodi ya upitiaji wa mfumo wa afya ya Taasisi ya chuo kikuu cha Duke, kwa namba+1-919-668-5111.

**TAMKO LA RIDHAA**

“Kusudi la utafiti huu, taratibu za kufuatwa, hatari na faida zimeelezwa kwangu mimi. Nimeruhusiwa kuuliza maswali niliyonayo, na maswali yangu yamejibiwa na nimeridhika. Nimekwisha ambiwa ni nani wa kuwasiliana nae kama nina maswali ya ziada. Nimesoma fomu hii ya ridhaa na nimekubali kushiriki katika utafiti huu kwa “kuelewa kwamba ninaweza kujitoa muda wowote. Nimeambiwa kwamba nitapewa nakala ya fomu ya ridhaa iliyosainiwa na kuandikwa tarehe niihifadhi.

Jina la mshiriki (print)                                                                                 Sahihi ya mshiriki na tarehe

##

**Questionnaires - English**

## AUD Screening questions – DSM-V (English):

A1. Had times when you ended up drinking more, or longer, than you intended?

A2. More than once wanted to cut down or stop drinking, or tried to, but couldn’t?

A3. Spent a lot of time drinking? Or being sick or getting over other aftereffects?

A4. Wanted a drink so badly you couldn’t think of anything else?

A5. Found that drinking—or being sick from drinking—often interfered with taking care of your home or family? Or caused job troubles? Or school problems?

A6. Continued to drink even though it was causing trouble with your family or friends?

A7. Given up or cut back on activities that were important or interesting to you, or gave you pleasure, in order to drink?

A8. More than once gotten into situations while or after drinking that increased your chances of getting hurt (such as driving, swimming, using machinery, walking in a dangerous area, or having unsafe sex)?

A9. Continued to drink even though it was making you feel depressed or anxious or adding to another health problem? Or after having had a memory blackout?

A10. Had to drink much more than you once did to get the effect you want? Or found that your usual number of drinks had much less effect than before?

A11. Found that when the effects of alcohol were wearing off, you had withdrawal symptoms, such as trouble sleeping, shakiness, restlessness, nausea, sweating, a racing heart, or a seizure? Or sensed things that were not there?

## AUDIT (English):

“Thank you for agreeing to participate in our study which makes sure these questions developed internationally work in Tanzanian Swahili and in Moshi. Please answer the following questions. “

| **B1. How often do you have a drink containing alcohol?**   1. Never [Skip to Q9-10] 2. Monthly or less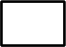 3. 2 to 4 times a month 4. 2 to 3 times a week 5. 4 or more times a week | **B6. How often during the last year have you needed a drinking in the morning to get yourself going after a heavy drinking session?**   1. Never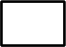 2. Less than monthly 3. Monthly 4. Weekly 5. Daily or almost daily |
| --- | --- |
| **B2. How many drinks containing alcohol do you have on a typical day when you are drinking?**   1. 1 or 2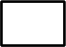 2. 3 or 4 3. 5 or 6 4. 7, 8 or 9 5. 10 or more | **B7. How often during the last year have you had a feeling of guilt or remorse after drinking?**   1. Never 2. Less than monthly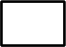 3. Monthly 4. Weekly 5. Daily or almost daily |
| **B3. How often do you have six or more drinks on one occasion?**   1. Never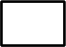 2. Less than monthly 3. Monthly 4. Weekly 5. Daily or almost daily   *Skip to Questions B9 and B10 if total Score for QB2 & B3=0* | **B8. How often during the last year have you been unable to remember what happened the night before because you had been drinking?**   1. Never 2. Less than monthly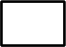 3. Monthly 4. Weekly 5. Daily or almost daily |
| **B4. How often during the last year have you found that you were not able to stop drinking once you started?**   1. Never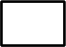 2. Less than monthly 3. Monthly 4. Weekly 5. Daily or almost daily | **B9. Have you or someone else been injured as a result of your drinking?**  (0) No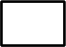  (2) Yes, but not in the last year  (4) Yes, during the last year |
| **B5. How often during the last year have you failed to do what was normally expected from you because of drinking?**   1. Never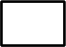 2. Less than monthly 3. Monthly 4. Weekly 5. Daily or almost daily | **B10. Has a relative or friend or doctor or another health worker been concerned about your drinking or suggested you cut down?**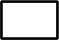  (0) No  (2) Yes, but not in the last year  (4) Yes, during the last year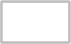  *B11: Total AUDIT SCORE HERE:* |

**C. Alcohol Use History**

C1. In the past month, typically how many times per week do you drink?

| Every day | 1 |
| --- | --- |
| Nearly every day | 2 |
| 3 or 4 times a week | 3 |
| Once or twice a week | 4 |
| 2 or 3 times a month | 5 |
| About once a month | 6 |
| 6-11 times a year | 7 |
| 1-5 times a year | 8 |
| No alcohol during last 12 months | 9 |
| Unknown | 99 |

**C2.** How much do you drink on a typical drinking occasion?

| Drink | % alcohol | Size of container | # containers |
| --- | --- | --- | --- |
| Low alcohol beer;  Yes/No | 2.5% |  |  |
| Beer ;                  Yes/No | 4.5% |  |  |
| Strong Beer;     Yes/No | 7.5% |  |  |
| Table Wine;      Yes/No | 11% |  |  |
| Fortified Wine:  Yes/No | 18% |  |  |
| Konyagi:             Yes/No | 35% |  |  |
| Spirits:                Yes/No | 40% |  |  |
| Strong Spirits:   Yes/No | 60% |  |  |
| Gongo                 Yes/No | 60% |  |  |
| Dadii;                 Yes/No | 5% |  |  |
| Mbege;              Yes/No | 5% |  |  |
| Dengerua;         Yes/No | 7.5% |  |  |

##

## Questionnaires – Swahili

##

## AUD Screening questions – DSM-V (Swahili):

A1. Imewahi kutokea wakati ukanywa Zaidi au ukanywa kwa muda mrefu kuliko ulivyotegemea?

A2. Imewahi kutokea ukajaribu Zaidi ya mara moja kusitisha au kuacha kunywa na ukagundua kwamba huwezi kufanya hivyo?

A3. Uliwahi kutumia muda mwingi kunywa au kuwa na hangover?

A4. Umewahi kuwa na hamu sana ya kunywa?

A5. Unywaji wako au hangover inakusababishia kushindwa kulea familia yako au nyumba yako au imekusababishia matatizo kazini au imekusababishia matatizo shuleni mara kwa mara?

A6. Uliendelea kunywa hata kama ilikusababishia matatizo na familia yako au marafiki?

A7. Umewahi kukata tamaa au kusitisha kufanya shughuli zako za muhimu au zinazokuvutia au zinazokufurahisha ili ukanywe?

A8. Imewahi kutokea zaidi ya mara moja wakati unakunywa au baada ya kunywa ukajiweka katika hali ambayo ingekuongezea uwezekano wa kuumia (kama vile kuendesha, kuogelea, kutumia mashine, kutembea kwenye maeneo hatarishi, au kufanya mapenzi ambayo sio salama)?

A9. Uliwahi kuendelea kunywa hata kama ilikufanya kuwa na huzuni au wasiwasi au kuongeza tatizo lingine la kiafya au ilikusababishia kupoteza kumbukumbu?

A10. Umewahi kunywa kiasi kikubwa cha pombe ili uweze kulewa, au umewahi kujikuta kwamba hukuweza “kulewa” kwa kiwango chako cha kawaida ambacho huwa kunywa?

A11. Umewahi kujikuta wakati pombe imeisha kichwani, ukapata dalili ya matokeo ya kuisha pombe kichwani kama kukosa usingizi, kutetemeka, kuwashawa, wasiwasi, kuhangaika, kichefuchefu, au kutokwa na jasho au kuhisi vitu ambavyo havipo?

**AUDIT (Swahili):**

“Ahsante kwa kukubali kushiriki kwenye utafiti wetu ambao unahakikisha maswali haya yameandaliwa kimataifa yatumike kwa kiswahili cha Tanzania na Moshi. “

“Sasa naenda kukuuliza baadhi ya maswali kuhusu matumizi yako ya pombe katika mwaka uliopita”.[*Eleza kama muhimu maana ya neno vinywaji vyenye kilevi kwa kutumia mifano ya pombe za kienyeji, mvinyo, vodka n.k. weka jibu kutokana na “vinywaji vyenye kiwango”weka namba ya jibu sahihi kwenye kisanduku kulia.* ]

Umewahi kunywa pombe? Hapana = 0 *NENDA HADI* B9,B10*

NDIYO, LAKINI SI KWA MWAKA ULIOPITA = 1 *NENDA HADI* B9,B10*

NDIYO, KWA MWAKA ULIOPITA = 2 Endelea B1

| **B1. Ni mara ngapi unakunywa kinywaji chenye kilevi??**   1. Haijawahi kutokea [Ruka hadi Q9-10] 2. Kila mwezi au chini ya mwezi 3. Mara 2 hadi 4 kwa mwezi 4. Mara 2 hadi 3 Kwa wiki 5. Mara 4 au zaidi kwa wiki | **B6.Ni mara ngapi katika mwaka uliopita ulihitaji kunywa pombe asubuhi ili uweze kuendelea kufanya mambo yako baada ya kikao kirefu cha kunywa pombe sana?**   1. Haijawahi kutokea 2. Chini ya kila mwezi 3. kila mwezi 4. Kila wiki 5. Kila siku au Karibu kila siku |
| --- | --- |
| **B2. Ni vinywaji vingapi vyenye kilevi ambavyo kwa kawaidaunakunywa kwa siku anapokuwa unakunywa??**   1. 1 au 2 2. 3 au 4 3. 5 au 6 4. 7, 8 au 9 5. 10 au zaidi | **B7. Ni Mara nagapi katika mwaka uliopita ulijihisi mwenye hatia au majuto baada ya kunywa?**   1. Haijawahi kutokea 2. Chini ya kila mwezi 3. kila mwezi 4. Kila wiki 5. Kila siku au Karibu kila siku |
| **B3. Ni Mara ngapi unakunywa vinywaji sita au zaidi kwa wakati mmoja/kikao kimoja?**   1. Haijawahi kutokea 2. Chini ya kila mwezi 3. kila mwezi 4. Kila wiki 5. Kila siku au Karibu kila siku   *Ruka mpaka swali B9 na B10 Kama jumla ya maksi ya QB2 & B3=0* | **B8.Ni Mara ngapi katika mwaka uliopita ulishindwa kukumbuka yaliyotokea kabla ya usiku kwa sababu ulikuwa amelewa?**   1. Haijawahi kutokea 2. Chini ya kila mwezi 3. kila mwezi 4. Kila wiki 5. Kila siku au Karibu kila siku |
| **B4. Ni mara ngapi katika mwaka uliopita uligundua kuwa hukuweza kuacha kunywa pombe mara tu alipoanza kunywa?**   1. Haijawahi kutokea 2. Chini ya kila mwezi 3. kila mwezi 4. Kila wiki 5. Kila siku au Karibu kila siku | **B9. Wewe au mtu yeyote mwingine amewahi kuumia kwa sababu ya kunywa kwako?**  (0) Hapana  (2) Ndiyo,Lakini si kwa mwaka uliopita  (4) Ndiyo,katika mwaka uliopita |
| **B5.Ni mara ngapi katika mwaka uliopita ulishindwa kufanya vitu vilivyotarajiwa kufanywa na wewe kwa sababu ya kunywa?**  (1) Haijawahi kutokea  (2) Chini ya kila mwezi  (3) kila mwezi  (4) Kila wiki  (5) Kila siku au Karibu kila siku | **B10. Kuna Ndugu au rafiki au Daktari au mfanyakazi mwingine wa afya amewahi kuguswa au kukushauri kuhusu kuacha pombe?**  (0) Hapana  (2) Ndiyo, Lakini si kwa mwaka uliopita  (4) Ndiyo, katika mwaka uliopita  *B11: Jumla ya MAKSI YA AUDIT HAPA:* |

**C.Historia ya matumizi ya pombe**

C1.Katika mwezi uliopita, kwa kawaida ni mara ngapi unakunywa

| Kila siku | 1 |
| --- | --- |
| Karibu kila siku | 2 |
| Mara 3 au 4 kwa wiki | 3 |
| mara moja au mara mbili kwa wiki | 4 |
| Mara 2 au mara 3 kwa mwezi | 5 |
| Ni kama mara moja kwa mwezi | 6 |
| Mara 6-11 kwa mwaka | 7 |
| Mara 1-5 kwa mwaka | 8 |
| Sijakunywa pombe katika miezi 12 iliyopita | 9 |
| Haijulikani | 99 |

**C2.**Ni kiasi gani unakunywa kwa kawaida wakati unakuwa unakunywa?

| Drink | % alcohol | Size of container | # containers |
| --- | --- | --- | --- |
| Low alcohol beer; Yes/No | 2.5% |  |  |
| Beer ; Yes/No | 4.5% |  |  |
| Strong Beer; Yes/No | 7.5% |  |  |
| Table Wine; Yes/No | 11% |  |  |
| Fortified Wine: Yes/No | 18% |  |  |
| Konyagi: Yes/No | 35% |  |  |
| Spirits: Yes/No | 40% |  |  |
| Strong Spirits: Yes/No | 60% |  |  |
| Gongo Yes/No | 60% |  |  |
| Dadii; Yes/No | 5% |  |  |
| Mbege; Yes/No | 5% |  |  |
| Dengerua; Yes/No | 7.5% |  |  |
