# Supplementary material for: Clinical evaluation of the alcohol use disorders identification test (AUDIT) in Moshi, Tanzania
Source: PLoS One. 2023 Nov 8;18(11):e0287835. doi: 10.1371/journal.pone.0287835 (PMC10631671; doi:10.1371/journal.pone.0287835)
Supplement: Attachment — Submitted filename: review audit tanzania.docx [file pone.0287835.s002.docx]

I thank you for the opportunity to read the article which deals with a very important area within alcohol research, namely validating measuring instruments in different cultures. The aim of this study was to validate the AUDIT and its subscales in Tanzania and also to investigate the predictive value of these. However I identified some deficiencies.

The sample was an availability sample of 259 people who were asked on the street to complete questionnaires, which means that it is difficult to generalize the results to others than those who were not asked.

On page 13, the authors define reliability and validity. The definition of validity is limited to conceptual validity. Reliability can also be defined as the relationship between different items in the scale.

To diagnose alcohol use disorder, a self-rating scale with 11 items based on DSM-5 was used. However, there is no information on the validity of the scale. To quantify standard drinks, the American norm for standard drink has been used. However, it may be difficult for the participants in the study to translate the amount of alcohol they drank into standard American drinks.

The data has been analyzed with confirmatory factor analysis, which is a hypothesis testing method, but there is no information about which hypothesis is being tested. It should be clearer that it is a hypothesis about a one-dimensional model that is tested with a confirmatory factor analysis. Convergent validity means a relationship between two measuring instruments, but the authors have not stated which instrument was used in the calculation. The authors write that 127 participants had AUD divided into 50 mild, 30 moderate and 72 severe. Then the total will be 152 participants.

In terms of sensitivity and specificity, the numbers in the text does not agree with what is stated in table 3.
